# Tsetse Control and Gambian Sleeping Sickness; Implications for Control Strategy

**DOI:** 10.1371/journal.pntd.0003822

**Published:** 2015-08-12

**Authors:** Inaki Tirados, Johan Esterhuizen, Vanja Kovacic, T. N. Clement Mangwiro, Glyn A. Vale, Ian Hastings, Philippe Solano, Michael J. Lehane, Steve J. Torr

**Affiliations:** 1 Liverpool School of Tropical Medicine, Liverpool, United Kingdom; 2 Bindura University of Science Education, Department of Animal Science, Bindura, Zimbabwe; 3 Southern African Centre for Epidemiological Modelling and Analysis, University of Stellenbosch, Stellenbosch, South Africa; 4 Institut de Recherche pour le Developpement (IRD), UMR IRD-CIRAD 177 INTERTRYP CIRDES 01, Bobo-Dioulasso, Burkina Faso; 5 Warwick Medical School, University of Warwick, Coventry, United Kingdom; Universidad Autónoma de Yucatán, MEXICO

## Abstract

**Background:**

Gambian sleeping sickness (human African trypanosomiasis, HAT) outbreaks are brought under control by case detection and treatment although it is recognised that this typically only reaches about 75% of the population. Vector control is capable of completely interrupting HAT transmission but is not used because it is considered too expensive and difficult to organise in resource-poor settings. We conducted a full scale field trial of a refined vector control technology to determine its utility in control of Gambian HAT.

**Methods and Findings:**

The major vector of Gambian HAT is the tsetse fly *Glossina fuscipes* which lives in the humid zone immediately adjacent to water bodies. From a series of preliminary trials we determined the number of tiny targets required to reduce *G*. *fuscipes* populations by more than 90%. Using these data for model calibration we predicted we needed a target density of 20 per linear km of river in riverine savannah to achieve >90% tsetse control. We then carried out a full scale, 500 km^2^ field trial covering two HAT foci in Northern Uganda to determine the efficacy of tiny targets (overall target density 5.7/km^2^). In 12 months, tsetse populations declined by more than 90%. As a guide we used a published HAT transmission model and calculated that a 72% reduction in tsetse population is required to stop transmission in those settings.

**Interpretation:**

The Ugandan census suggests population density in the HAT foci is approximately 500 per km^2^. The estimated cost for a single round of active case detection (excluding treatment), covering 80% of the population, is US$433,333 (WHO figures). One year of vector control organised within the country, which can completely stop HAT transmission, would cost US$42,700. The case for adding this method of vector control to case detection and treatment is strong. We outline how such a component could be organised.

## Author Summary

Sleeping sickness is controlled by case detection and treatment but this often only reaches less than 75% of the population. Vector control is capable of completely interrupting HAT transmission but is not used because of expense. We conducted a full scale field trial of a refined vector control technology. From preliminary trials we determined the number of insecticidal tiny targets required to control tsetse populations by more than 90%. We then carried out a full scale, 500 km^2^ field trial covering two HAT foci in Northern Uganda (overall target density 5.7/km^2^). In 12 months tsetse populations declined by more than 90%. A mathematical model suggested that a 72% reduction in tsetse population is required to stop transmission in those settings. The Ugandan census suggests population density in the HAT foci is approximately 500 per km2. The estimated cost for a single round of active case detection (excluding treatment), covering 80% of the population, is US$433,333 (WHO figures). One year of vector control organised within country, which can completely stop HAT transmission, would cost US$42,700. The case for adding this new method of vector control to case detection and treatment is strong. We outline how such a component could be organised.

## Introduction

Human African trypanosomiasis (HAT = sleeping sickness) is a fatal disease caused by two separate parasites, *Trypanosoma brucei gambiense* and *T*. *b*. *rhodesiense*. HAT, along with its tsetse fly (*Glossina* spp.) vectors, is restricted to sub-Saharan Africa. The two forms of HAT are normally fatal if untreated and have distinct epidemiologies. Gambian HAT is a chronic disease of several years’ duration and is classically considered to be transmitted from person to person by ‘riverine’ tsetse. It is normally controlled by active or passive case detection and treatment with vector control playing little or no part. Gambian HAT comprised >98% of all officially reported HAT cases in 2009 [1]. Rhodesian HAT is an acute disease lasting months and is a zoonosis with a large number of domestic and wild animals acting as reservoirs and the vectors are generally ‘savannah’ tsetse. Vector control is central to curbing outbreaks of Rhodesian HAT and case screening is only carried out for humanitarian reasons [2,3]. Rhodesian HAT comprised <2% of all officially reported HAT cases in 2009 [1].

There were large-scale epidemics of Gambian HAT in the first half of the 20^th^ century which were largely brought under control by the 1960s through large-scale programmes of active case detection and treatment. The low number of cases at that time led to the neglect of the disease. That resulted in resurgence and another large-scale outbreak in the 1990s with 30,000 officially reported cases annually, considered to represent up to 300,000 people infected and left largely untreated in the field [4]. Since then the prevalence has fallen to historically low levels. The number of cases officially reported to WHO fell below 10,000 in 2009 [1] and has stayed there. It is speculated that the under-reporting ratio has fallen from 10:1 in the 1990s to about 3:1 suggesting there are currently about 30,000 cases annually [1]. It is recognised that these historically low levels of the disease offer an ideal opportunity to push for its elimination [5].

There is no vaccine for use against HAT. New diagnostics and an oral drug for stage 2 disease are expected to be in place in the next two years which will improve the situation. However, presently patient treatment is complicated by painful and invasive diagnostics, drug toxicity and the complexity of case management which is difficult to achieve in the remote, resource-poor rural settings where the disease is normally found [3]. Despite this, the major intervention against Gambian HAT remains the use of case detection and treatment programmes. This is undoubtedly successful in reducing infection levels and controlling outbreaks as well as preventing people who are already infected from dying. However there are well recognised drawbacks to this approach. One is that it fails to protect people from becoming infected, vector control remains the only means of doing this. A further major drawback of active case detection and treatment programmes is that on average they only cover about 75% of the population [6] in addition to which diagnostic sensitivity is low [7] so that they always leave a residual level of infection in the community (sometimes >50% [6]) which plays a critical role in sustaining transmission [8]. In addition, animal reservoirs may play more of a role in Gambian HAT than believed up to now [9]. Given these drawbacks it is odd that vector control is not more widely used as it can rapidly achieve complete interruption of transmission [10] and the slow reproductive rate of tsetse makes it unusually susceptible to insecticide-based methods of control. Certainly if the disease were present in Europe or the USA vector control would be a major element in control. The arguments put forward for not using it in Africa are that it is too costly and difficult to deliver in the remote, resource-poor settings where Gambian HAT flourishes.

Given the above we have been working on the development of cheaper, easier-to-deploy systems of tsetse control appropriate for use at the scale of a HAT focus. By reference to the HAT atlas [11] and the projected distribution of tsetse flies [12], we predict that more than 90% of Gambian HAT is transmitted by *G*. *fuscipes* sp. and most of the rest by *G*. *palpalis* sp. [13]. Consequently we have concentrated efforts on studying these species. The breakthrough was the surprising finding that these and other riverine species all respond well to extremely small (50 X 25 cm) insecticide-treated targets (‘tiny targets’) [14–18] which is in striking contrast to the more thoroughly studied savannah flies [19]. As described in this paper, these tiny targets are highly efficient in reducing the density of tsetse flies and we argue that they are easier and cheaper to deploy than conventional tsetse control devices.

There is currently a debate on how best to capitalise on the existing historically low levels of HAT with the aim of eliminating the disease. As a contribution to this debate we describe here small-scale trials on islands (<1km^2^) in Lake Victoria followed by a full scale, 500 km^2^ field trial covering two HAT foci in Northern Uganda to determine the efficacy of tiny targets. We consider costs and, in the light of the results, we propose that vector control can play a very significant role, alongside case detection and treatment, in efforts to eliminate Gambian HAT.

## Materials and Methods

### Kenya

To determine the number of targets required to control *G*. *f*. *fuscipes* a series of trials was first performed on the following islands in the Kenyan section of Lake Victoria: Big Chamaunga (-0.426° latitude, 34.233° longitude; surface area≈0.2km^2^; circumference≈1.5 km), Small Chamaunga (-0.431° latitude, 34.227° longitude; surface area≈0.2km^2^; circumference≈1km), Manga (-0.353° latitude, 34.253° longitude; surface area≈1km^2^, circumference≈2km) and Magare (-0.348° latitude, 34.262° longitude, surface area≈2km^2^, circumference≈2.5km). *G*. *f*. *fuscipes* is the only species of tsetse present on the islands and lake shore. Each island had typical lake shore vegetation consisting predominantly of clumps of freshwater mangrove trees (*Aeschynomene eraphroxylon*) intermixed with *Dombeya spp*. and *Lantana camara* stands. Apart from occasional visits from fishermen, Big Chamaunga and Small Chamaunga are uninhabited. Tsetse populations are sustained by wild hosts, particularly Nile monitor lizard (*Varanus niloticus*). Manga and Magare are inhabited and potential hosts include cattle, humans, hippopotamus and monitor lizard. For a limited period, we also monitored tsetse on the shores of Lake Victoria within 1 km of Uyoma (-0.346° latitude, 34.273° longitude), Magare lies ~1 km offshore from Uyoma. The shore of Lake Victoria in the vicinity of Uyoma comprises fishing villages intermixed with subsistence farms and natural lakeshore vegetation. Potential hosts include humans, cattle, hippopotamus and monitor lizard.

#### Island trial 1 (20 targets/km; January 2011-December 2012)

In the first trial, we assessed the impact of deploying targets at 50m intervals along the shore of Big Chamaunga. Small Chamaunga was used as the control (non-intervention) area. The two islands are ~500m apart with Small Chamaunga being closer to the lake shore (~150m distant).

#### Island trial 2 (10 targets/km; October 2012-December 2013)

In a second trial, targets were deployed at 100m intervals along the shore of Manga island and Magare served as the control (non-intervention).

#### Tiny targets

**Fig 1:**
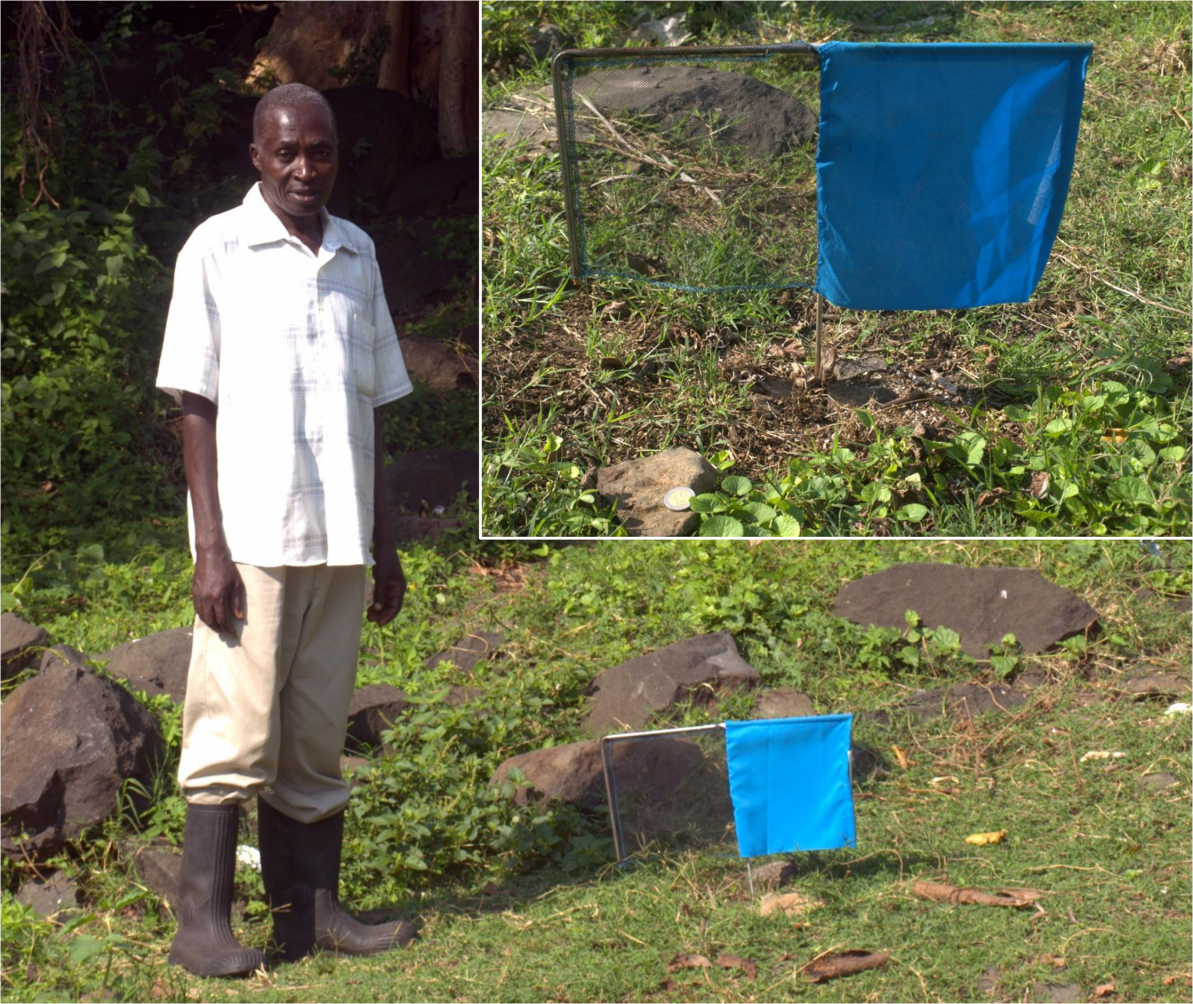
A tiny target deployed near the lakeshore for control of *G*. *f*. *fuscipes* on Big Chamaunga island, Kenya.

Tiny targets (Fig 1) were manufactured by Vestergaard-Frandsen (Lausanne, Switzerland) and were made of pthalogen blue polyester cloth (25x25cm) attached to fine (150 denier) black polyethylene mosquito netting (25x25cm) impregnated with deltamethrin (300 mg/m^2^). Vegetation was cleared within 2m of the target. Using a simple steel or wooden frame, targets were deployed with the bottom edge ~10cm above the soil surface (Fig 1). The locations of targets were recorded using a global positioning system (GPSmap 78, Garmin (Europe) Ltd., Southampton, UK). A weekly record was kept of target condition and lost or damaged targets were noted and replaced.

#### Tsetse monitoring

For tsetse monitoring, biconical traps [20] were deployed, between 200 and 300m apart, along the lakeshore of all islands. Four traps were deployed on Small Chamaunga and Big Chamaunga and five on each of Manga and Magare. For Big Chamaunga and Manga only, an additional trap was placed at the centre of the island. Riverine tsetse, as their name suggests, are concentrated within the vegetation fringing lakes and rivers and hence monitoring efforts were concentrated there. The additional trap deployed in atypical habitat at the centre of two islands allowed us to assess whether tsetse persisted there after the deployment of targets. Traps on the islands were operated between 08:00h and 17:00h daily, Monday to Friday, providing ~20 collections/site/month. At Uyoma, five traps were deployed at ~200m intervals along the lakeshore and provided ~8 collections/site/month.

In addition to biconical traps, we also used electrified targets (e-targets) to monitor the tsetse population. The e-target was based on the Standard target used previously [16] and comprised a 25x25cm panel of Phthalogen blue cotton flanked by an equal-sized panel of fine black polyester netting (Quality no. 166, Swisstulle, Nottingham, UK). The cloth and netting were then covered with an electric grid of fine (0.2 mm dia) copper wires, 8mm apart. Tsetse contacting the target were killed and fell into a tray of water placed below the e-target where they were retained. The catch from the e-target therefore provided a measure of the number of tsetse killed by an insecticide-treated tiny target. E-targets catch 2–5x more *G*. *f*. *fuscipes* than a biconical trap and hence provide a more sensitive measure of tsetse abundance. The e-target was operated between 08:00h and 12:00h for ~20 d/month. The e-target was operated at each site where tiny targets were usually placed following a randomisation procedure that ensured that over the course of 20 days, an e-target was operated at all sites where tiny targets were present. On a day when the e-target was deployed the tiny target was removed temporarily.

The fact that the islands and surrounding area of Mbita is free of HAT [11] permitted us to use a third method of tsetse monitoring–a human flyround. To estimate the number of tsetse landing on a human, a matt black panel (30x30cm) covered with ‘sticky film’ (Rentokil FICS mk1, Barrettine, Bristol, UK) was carried on the back of a research assistant for 2x30 minute periods, commencing at 09:00h and 12:00h, following a predetermined route that followed the entire shore of the island. Flyrounds were also conducted for ~20 days each month.

#### Effective life of the targets

The knockdown of tsetse exposed to tiny targets was assessed using field-caught *G*. *f*. *fuscipes* within 5km of Mbita beside the shore of Lake Victoria and *G*. *pallidipes* at Rekomitje field station in the Zambezi Valley of Zimbabwe using standard methods. Tiny targets were exposed to weathering and bioassayed at intervals for up to 11 months.

To assess whether the colour in blue cloth faded on weathering, experiments were conducted near Mbita to compare catches from e-targets made from new (i.e., unexposed) or old cloth and net taken from tiny targets that had been exposed for 6–12 months on Big Chamaunga. These two types of target were compared with a Standard e-target as used in previous studies of tiny targets [16] and as a monitoring tool in the present study. The three types of target were compared using a Latin square design of targets x sites x treatments. Sites were >100 m apart and the comparison was repeated for 18 daily replicates.

### Uganda

A large-scale trial of tiny targets was conducted between September 2011 and December 2013 in the adjoining HAT foci of Arua and Maracha. The adjoining Koboko HAT focus was used as a control where no targets were deployed. All three HAT foci are in northern Uganda where HAT has been a long-standing health problem. The study area comprises farms cultivating a variety of crops including cassava, millet, peanuts, matoke, sesame and tobacco. The numbers of livestock (cattle, pigs, goats) were low. The area comprises not only perennial rivers such as the Enyau and Kochi but also many small (<3m wide) tributaries and seasonal streams which dried out during the dry season (January-March). Along the river banks are narrow (~2–5m) bands of natural riverine vegetation which included *Cynometra alexandri*, *Entada abyssinica*, *Acacia seyal*, *Ekebergia capensis*, *Plectranthus barbatus* and *Schrebera alata*. In general, the upstream sections which dry out seasonally have narrower bands of riverine vegetation and are expected to sustain smaller populations of tsetse, especially during the dry season. Apart from monitor lizards wild animals are rare, and hence lizards, humans and domestic cattle are likely to be the main hosts of tsetse. *G*. *f*. *fuscipes* was the only tsetse species caught during this study.

**Fig 2:**
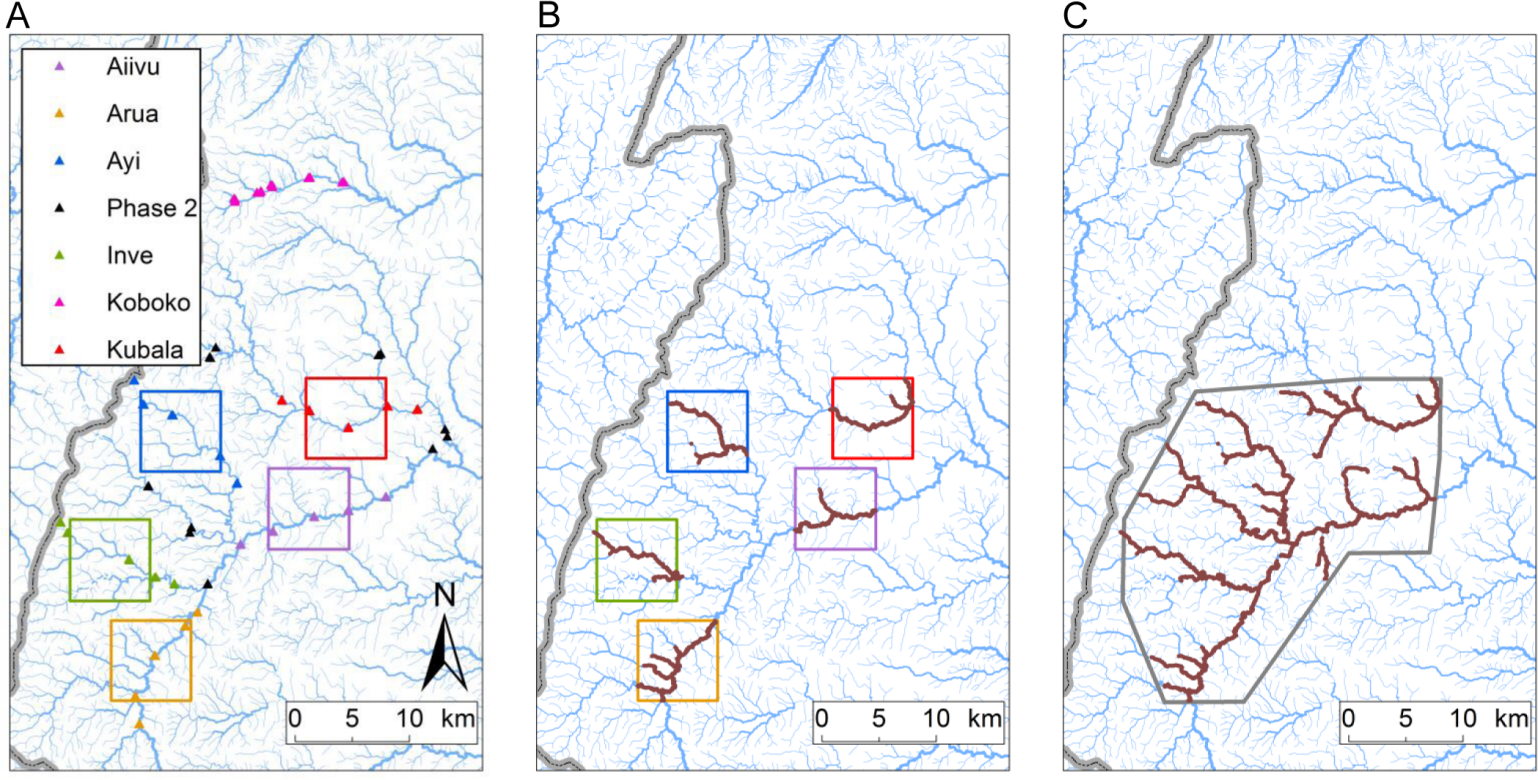
Locations of traps and tiny targets in NW Uganda. (A) Locations of monitoring traps for each intervention block (7 x 7 km squares) and rivers referred to in text (Enyau, Oluffe and Kochi). (B) Locations of targets (brown sections of rivers) within the five intervention blocks in 2013 (Ku = Kubala, Ay = Ayi, Ai = Aiivu, In = Inve, Ar = Arua). (C) Location of targets during the second phase of the trial; grey-coloured polygon denotes the extent of the operational area during Phase 2.

The trial comprised two phases. In Phase 1 (November 2011–December 2012), targets were deployed in five separate blocks, each 7x7km square to produce an intervention area of 50km^2^ per block and a total intervention area of 250km^2^ (Fig 2). In Phase 2 (December 2012–December 2013), targets were deployed over an area of about 500km^2^. The area treated in Phase 2 was formed by a 500 km^2^ polygon that included the five original Phase 1 blocks (Fig 2). Phase 1 simulated a community-based intervention which could be led by a small group of villages. Phase 2 simulated a larger operation that might be implemented by government or non-governmental agencies. The impact of targets was assessed by comparing the apparent density of tsetse in the intervention areas with sites in an adjacent District, Koboko, where no targets were deployed.

Targets were deployed in areas within Arua and Maracha Districts, extending between 3.053°–3.310° N and 30.838°–31.082° E (Fig 2). The non-intervention control area in Koboko District extended along the Kochi river between 3.451°–3.465° N and 30°58’–31°03’E (Fig 2). Apart from monitor lizard, wild hosts are rare and hence lizards, humans and domestic cattle are likely to be the main hosts of tsetse. *G*. *f*. *fuscipes* was the only tsetse species found in the study areas [21,22].

#### Preliminary entomological survey

Between October and December 2010, the apparent density of tsetse was assessed at 237 sites across Arua, Maracha and Koboko Districts using pyramidal traps [23]; pyramidal traps are routinely used by District tsetse control teams in Uganda and so we used these rather than biconical traps. Traps were deployed largely along rivers and streams, where *G*. *f*. *fuscipes* concentrate, but ~10% of sites were located in farming areas away from river courses. Catches were collected from traps at 24h intervals for a median of 3d (range, 1–8d).

#### Phase I: Small-scale intervention (November 2011–December 2012)

Five 50km^2^ blocks (‘Arua’, ‘Inve’, ‘Aiivu’, ‘Ayi’ and ‘Kubala’) were identified within the polygon of 500 km^2^, (Fig 2B) and 1,336 tiny targets were deployed between November and December 2011 at a density of ~20 targets per linear km, equating to a density of approximately 5.3 (1,336/250) tiny targets per km^2^. All the targets were replaced with new ones in May-June 2012.

#### Phase II: Large-scale intervention (November 2012–December 2013)

Between November 2012 and January 2013 the intervention was extended to a single 500km^2^ block by replacing the original 1,336 and adding a further 1,536 new tiny targets (Fig 2C). Density was retained at ~20 targets per linear km and the operational area was ~500km^2^, giving an overall density of ~5.7 (2,872/500) targets/km^2^.

### Entomological monitoring

#### Phase 1 (7x7km blocks)

Pyramidal traps were deployed in pairs, 100m apart, at the (i) centre, (ii) edge and ~3km outside each block (Fig 2A). Entomological monitoring started in September 2011. Tsetse caught in the traps were collected and counted at 24h intervals, four times per week, weekly (~20 samples/trap/month) until January 2013 and fortnightly (~10 samples/trap/month) thereafter.

#### Phase 2 (500km^2^)

In January 2013, an additional 12 pyramidal traps were added to the existing ones, so the impact of the intervention in the whole 500km^2^ polygon could be assessed (Fig 2A). Following deployment of traps, catches were collected and counted at 24h intervals, four times per week, every second week (~10 samples/trap/month) until 30 November 2013.

#### Non-intervention area

Fifteen sites were located in the non-intervention area, ~3.5km apart (Fig 2A). Each site contained two traps, ~100m apart. Tsetse caught in the traps were counted at 24h intervals, four times per week, weekly until January 2013 and fortnightly thereafter.

### Statistics and models

#### Apparent densities of tsetse

Catches (*n*) from traps, e-targets and flyrounds were normalized and variances homogenized using a log_10_(*n*+1) transformation. Detransformed mean catches are reported accompanied by their 95% Confidence Intervals. Results from comparisons of old and new targets were subjected to analysis of variance and the significance of differences between means was assessed by Tukey’s Honest Significant Difference (HSD) test using R [24].

#### Analytical model of the level of tsetse control required to interrupt HAT transmission

To calculate the proportion of tsetse which need to be killed to stop transmission we re-evaluated a published model [10]. We selected the parameter values used by the original authors which are: Number of humans = 300; probability that a tsetse fly will feed on a human during a 3d period = 0.105; probability that a fly will acquire an infection if feeding on an infected human = 0.1; probability that a susceptible human will acquire an infection if bitten by an infected fly = 0.63; average duration of HAT incubation in a human prior to becoming infective = 12d; average duration of trypanosome incubation in tsetse before the fly become infectious = 25d; average human lifespan = 50 years; average tsetse lifespan = 45d.

#### Simulation models

The impact of targets on tsetse populations was modelled using Tsetse Muse, a deterministic simulation model of the distribution and abundance of a tsetse population [25] using the parameter values shown in supplementary materials (Table A in S1 Text). The model itself can be downloaded at www.tsetse.org. To model the impact of targets on island populations of tsetse, we used the ‘isolated population’ settings in which tsetse movement in and out of the operational area does not occur. To model the impact of targets on riverine populations, we used the ‘non-isolated population’ settings. The simulated population comprised a series of 60 contiguous cells arranged linearly, with each cell representing a 1km length of riverine habitat. Outputs showed the population density, age structure and composition of tsetse for each cell along the 60km of a simulated river. Simulations were made of operations in which targets were deployed in seven contiguous cells, simulating Phase 1 of the Uganda trial. To simulate Phase 2, where the operational area was expanded, the Phase 1 block (seven cells) was expanded to a longer section of 21 contiguous cells, with the original block of seven cells at its centre. For Phase 1 simulations, the predicted densities at the ‘centre’ and the ‘edge’ refer to the central and penultimate cell of the seven. Across the 60-cell stretch of simulated river, the central cell was at cell number 31.

## Results

### Kenya

#### Big Chamaunga, Kenya: Targets deployed at 50 m intervals (January 2011-December 2012)

**Fig 3:**
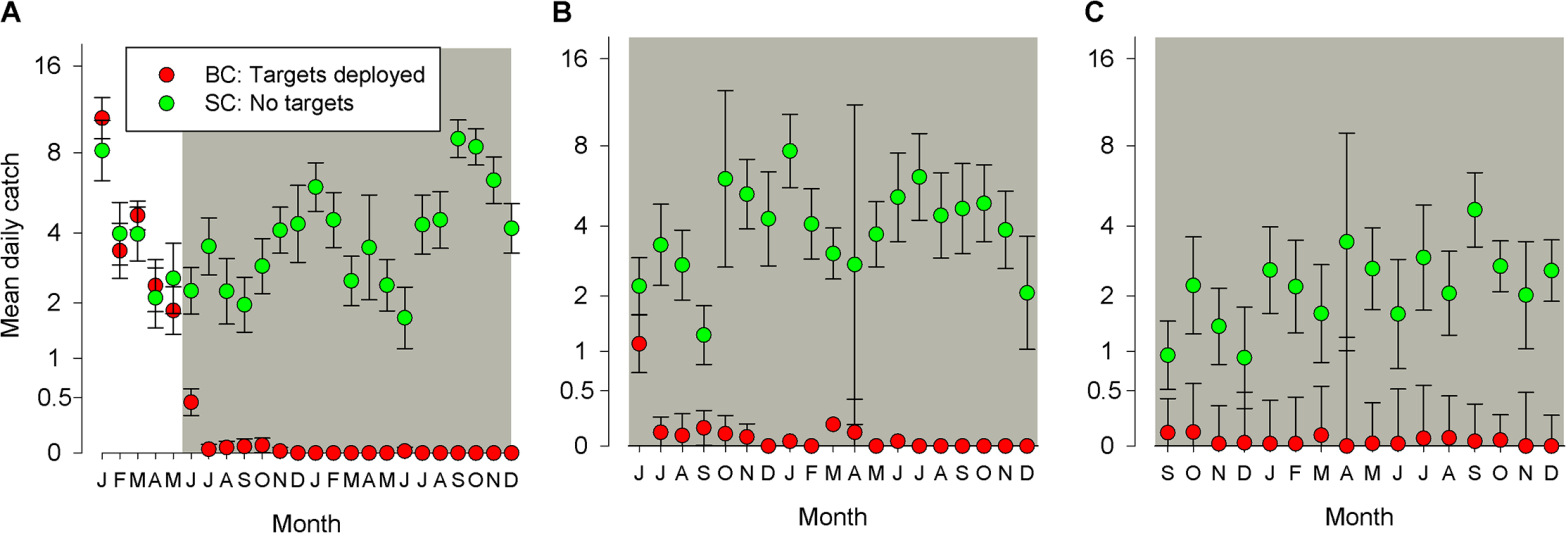
Mean daily catch (±95%CI) of tsetse from (A) traps, (B) E-targets and (C) flyrounds on islands with or without targets. Grey area indicates months when target were present on Big Chamaunga (BC); no targets deployed on Small Chamaunga (SC). Traps were operated before (Jan-May 2011) and after (June 2011 onwards) targets were deployed. E-targets (June 2011 onwards) and flyrounds (September 2011 onwards) were operated only after targets were deployed.

*Trap catches*. For the period January-May 2011, prior to the deployment of targets, the mean daily catch from traps operated on the lake shore of Big Chamaunga was 3.9 tsetse/trap/day (3.58–4.33, 95%CI) compared to 3.8 (3.27–4.29) for Small Chamaunga (Fig 3A). Following the deployment of targets on Big Chamaunga only, the mean catch there declined to 0.4 tsetse/trap/day in June and <0.1 tsetse/trap/day thereafter. By contrast, mean daily catches from traps deployed on Small Chamaunga remained >1 and showed no marked or persistent decline during 2011–12 (Fig 3). There was a seasonal decline in catches during the hot season from January to March but the population recovered in the ensuing wet season. A similar seasonal decline is apparent in the catches from Small Chamaunga in the following year (Jan-May 2012). The total catches of tsetse from Small Chamaunga during the 13 months between December 2011 and December 2012 were, respectively 5,406 (498 trap-days) compared to just two (992 trap-days) from Big Chamaunga. The trap operated at the centre of Big Chamaunga caught one fly (490 trap-days, May 2011-December 2012) in the period following the deployment of targets compared to 82 from the lakeshore traps. *E-targets and fly-rounds*. The overall level of catches from E-targets operated on Big Chamaunga and Small Chamaunga from June 2011 onwards was in general accordance with the catches from traps. On Big Chamaunga, the mean daily catch from an E-target was consistently <0.1 for the period June 2011–December 2012 and for nine months over this period, no tsetse were caught by the E-target (Fig 3B). By contrast, the daily mean catch of an E-target operated on Small Chamaunga was 3.8 (3.45–4.14, 95%CI). The total catch of tsetse from Big Chamaunga in the period December 2011–December 2013 was 8 tsetse (249 target-days) compared to 1,373 (246 target-days) from Small Chamaunga.

Fly-rounds commenced on Big Chamaunga and Small Chamaunga from September 2011 onwards and also showed a large (>10x) difference in catches. The mean daily catches for Small Chamaunga varied between 0.9 and 4.6 compared 0–0.1 on Big Chamaunga (Fig 3C), and the respective total catches in the period December 2011–December 2012 were 653 on Small Chamaunga, where targets were absent, compared to 26 on Big Chamaunga where they were present.

#### Manga and Magare Islands, Kenya: Targets at 100m intervals (Jan 2012-October 2013)

**Fig 4:**
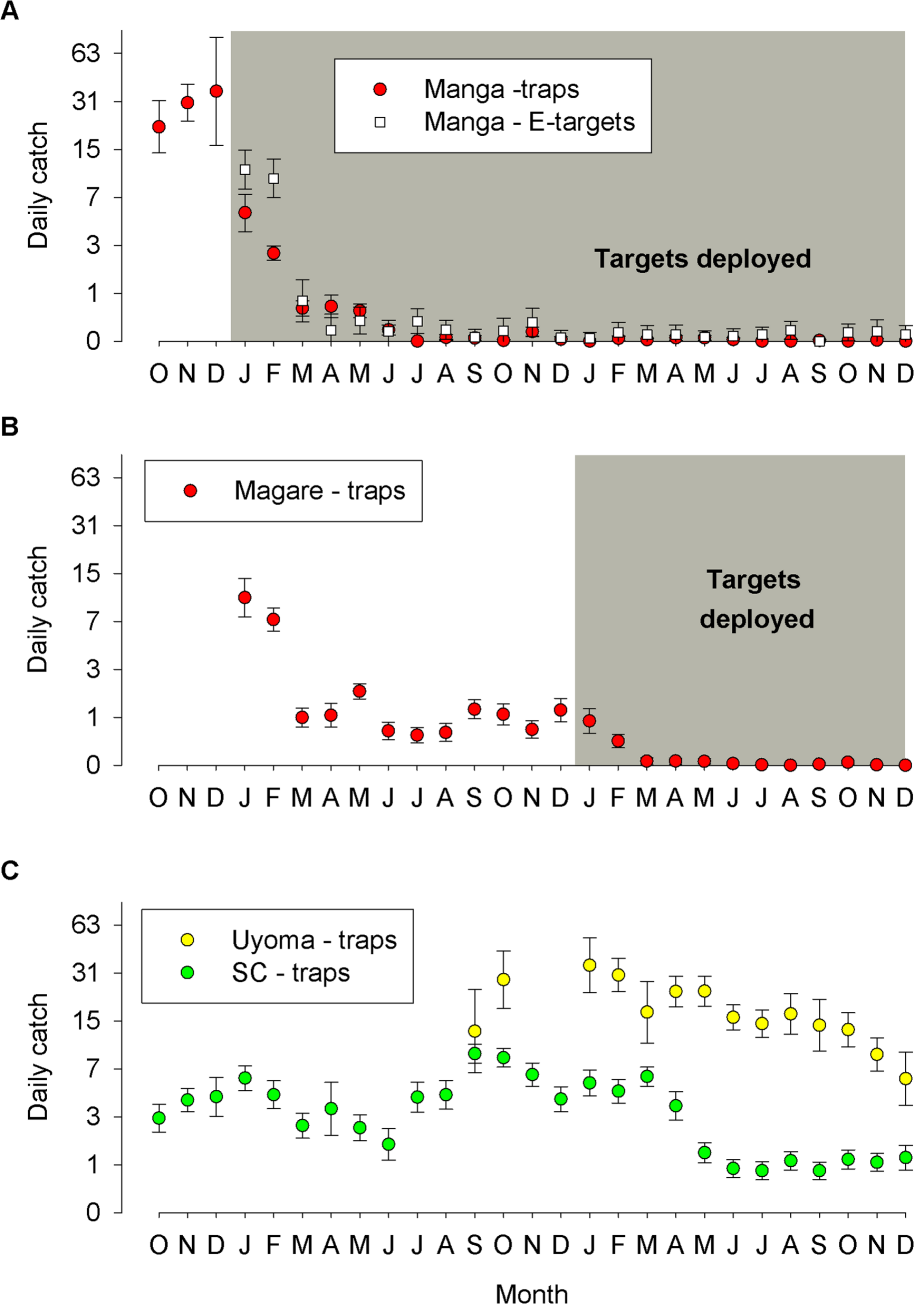
Mean daily catches of tsetse (±95%CI) from (A, B) sites where targets were present or (C) absent. Grey areas denote when targets were present on the islands of Manga and Magare. No targets were deployed on the island of Small Chamaunga (SC) or the mainland site of Uyoma (U). Sampling was carried out between October 2011 and November 2013.

Prior to the deployment of targets, the mean daily catch of tsetse from traps operated on Manga was 28.2 tsetse/trap/day (22.44–35.31, 95%CI). Following deployment of targets in January 2012, catches declined to 5.4 in January, 2.6 in February and continued at <1 tsetse/trap/day thereafter (Fig 4A). In contrast to the results from Big Chamaunga during 2012, a few tsetse were caught from traps in most months, there being only one month (July 2012) when no tsetse were caught from traps operated on Manga. Following the deployment of targets, the trap at the centre of Manga caught no tsetse (222 trap days) compared to 1532 flies (1110 trap days) from traps on the lake shore. E-targets were operated on Manga only from January 2012 onwards, when tiny targets were first deployed. The catches from the E-target showed a similar decline to trap catches: in January 2012 the mean daily catch from an E-target was 10.9 tsetse/target/day but was <1 from March onwards (Fig 4A).

Traps were operated on Magare from January onwards. Despite no targets being deployed there, catches declined from 10.3 tsetse/trap/day in January to ~1 tsetse/trap/day (Fig 4B). The decline on Magare might indicate a general decline in tsetse populations. However, there was no indication of a 90% decline in catches on the nearby Small Chamaunga during the same period (Fig 4C). The most likely explanation for the decline in catches on Magare is that targets on Manga also affected the population on Magare; the channel between the two islands is ~50m at its narrowest point and hence some movement of tsetse between the islands seems likely. Such movement might also explain why the population on Manga was not reduced to the very low levels observed on Big Chamaunga.

In January 2013, 21 targets were deployed at 100m intervals (i.e., the same density as used on Manga) along the shoreline of Magare. In the month immediately prior to deployment, the mean daily catch on Magare was 1.2 tsetse and this declined to 0.02 in March 2013 and remained at <0.1 tsetse/trap/day. During this period, traps operated at Uyoma, a site on the lake shore ~1km from Magare, caught ~10 tsetse/trap/day before targets were deployed on Magare and ~25 tsetse/trap/day after (Fig 4C). Following the deployment of targets on Magare, catches from traps and E-targets on Manga also declined to <0.1 tsetse/trap/day in accordance with the hypothesis that populations on Magare and Manga are not isolated from each other.

#### Effective life of targets

The blue panel showed no fading and consequent loss of attractiveness to tsetse throughout a 12 month exposure period in the field (Table B in S1 Text). The blue panel retained insecticidal activity throughout eight months but the efficacy of netting exposed for one month or longer was variable (Fig A in S1 Text). For netting, mean percentage knockdown varied between 37% (Month 5) and 90% (Month 3) for the first five months but was <30% (0–23%) for samples exposed for six months or longer. For netting exposed for relatively short periods (1–4 months) knockdown ranged widely: monthly maxima for Months 1–4 ranged between 91% and 100% whereas the minima were 10%–73%.

#### Modelling impact of targets on island populations of tsetse

**Fig 5:**
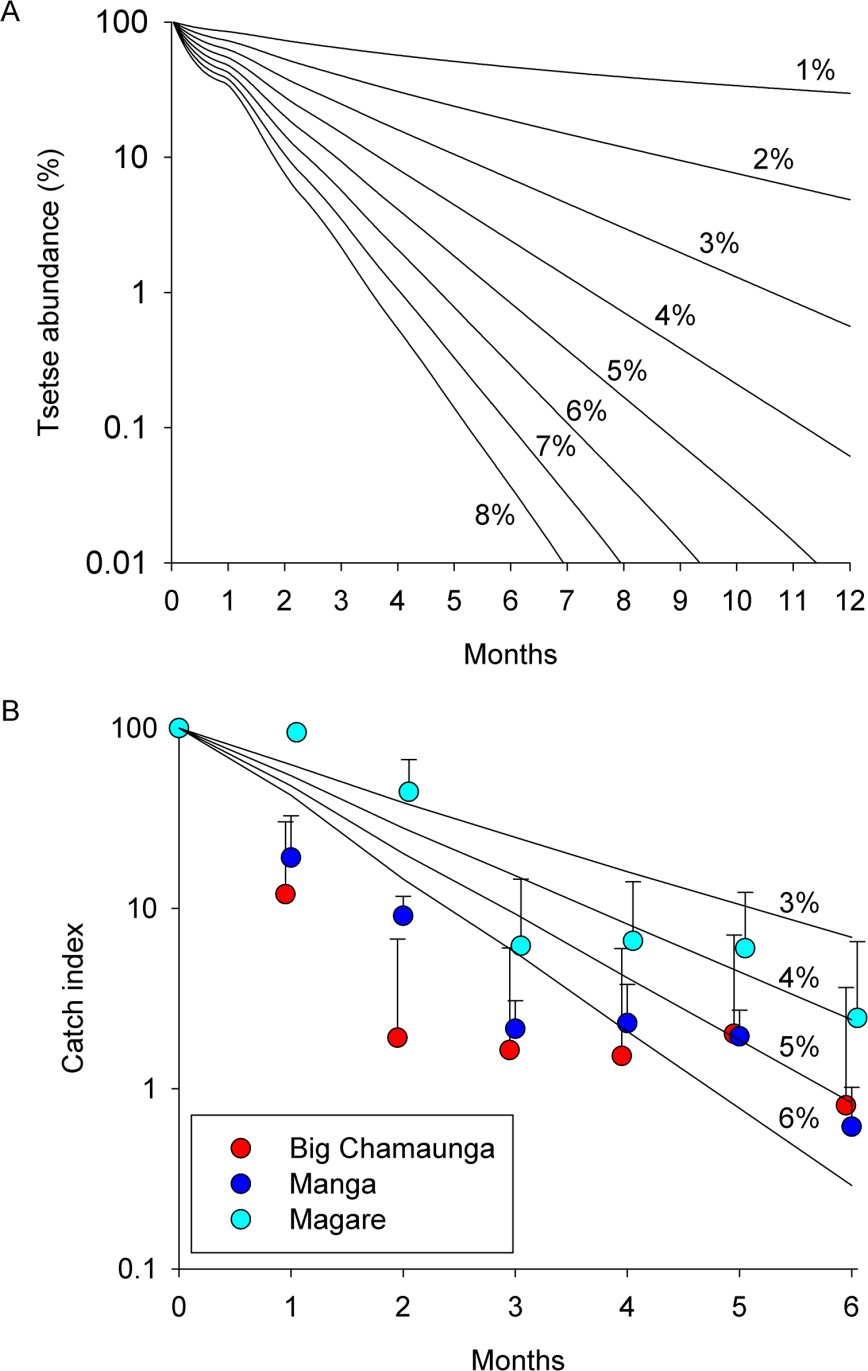
A. Predicted decline in a tsetse population over 12 months assuming imposed mortalities of 1–8%/day and (B) observed decline over six months on islands where tiny targets were deployed. A. For graph A, predicted declines were generated using the simulation model Tsetse Muse. For graph B, the Catch Index is the mean daily catch (+LSD) from traps for each month post-deployment of targets (Months 1–6) expressed as a percentage of the mean catch for the three months prior to deployment (Month 0). The pre-deployment mean catches were 3.1 (2.76–3.48, 95%CI) for Big Chamaunga, 28.2 (22.44–35.31) for Manga and 0.9 (0.79–1.12) for Magare. Lines show declines predicted over six months assuming imposed daily mortalities of 3–6%.

Modelling the effect of imposing daily mortalities of 1–8% on a tsetse population (Fig 5A) shows that imposing a mortality >3% is expected to eliminate a population in accord with several previous studies [25–27]. Defining the point where a population is eliminated is difficult without prolonged and extensive sampling of a population extending over many months [28]. However, to gain a rough indication of the daily mortality imposed on tsetse populations, we compared the percentage decline in catches over six months following the deployment of targets against the decline predicted by Tsetse Muse. The results (Fig 5B), show that the observed declines over six months on Manga were in rough accord with an imposed daily mortality of ~4% per day with an indication that higher densities of targets used in the trial on Big Chamaunga achieved a higher daily mortality approaching 6%.

### Uganda—West Nile, Uganda: October 2010–November 2013

#### Preliminary entomological survey (October—December 2010)

The mean catch of tsetse across all sites sampled (*n* = 235) was 2.4 tsetse/trap/day (1.89–2.98, 95% CI). Most traps (169/197 = 84%) deployed in riverine habitats caught tsetse (median catch = 2.7 tsetse/trap/day, range = 0–114) whereas most traps (36/38 = 95%) deployed in non-riverine habitats caught none (median = 0, range = 0–1).

#### Phase 1: Targets deployed in 7 x 7 km square blocks

**Fig 6:**
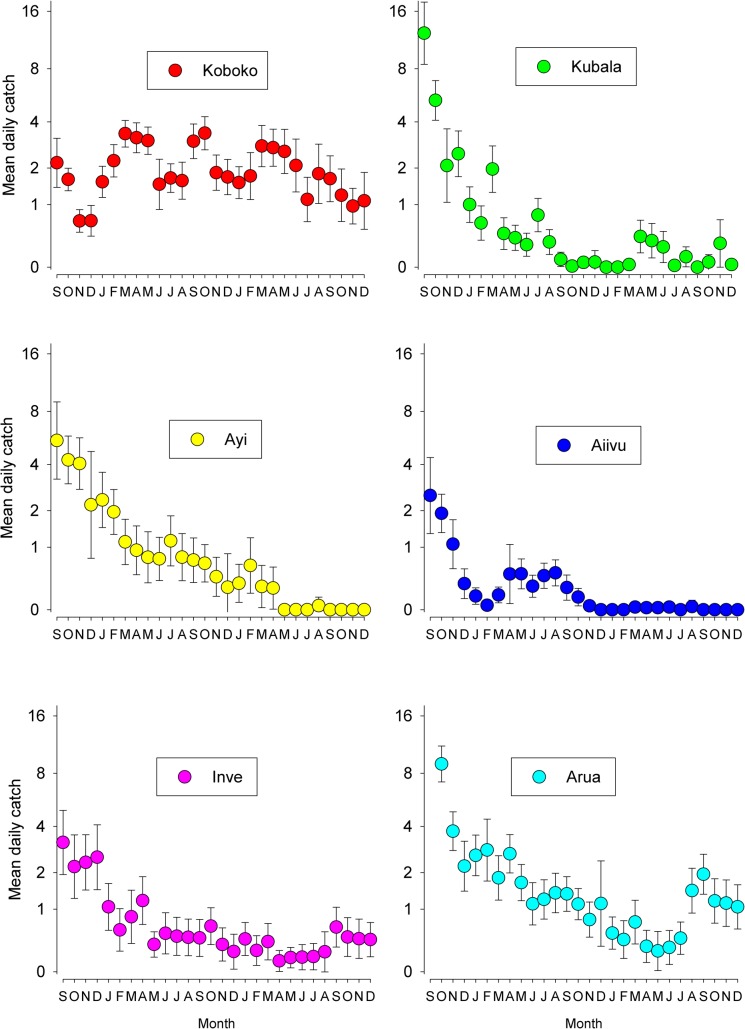
Daily mean catch (±95%CI) from traps operated in areas where tiny targets were present or absent (Koboko only) from November onwards.

Regular monitoring of tsetse within the prospective intervention and control (non-intervention) blocks began in September 2011 and targets were deployed within the five intervention blocks from November onwards. The results (Fig 6) show that for the two months prior to the deployment of targets, mean daily catches from Koboko (non-intervention block) was 2 tsetse/trap/day and 2–9 in the prospective intervention blocks. Using the data from the Island trials (above), we decided to place targets at 50 m intervals along the rivers in the intervention blocks. Following the deployment of targets, catches declined in the intervention blocks but remained steady in Koboko where targets were not deployed. Hence from June onwards, catches from traps within four of the intervention blocks were consistently <1 tsetse/trap day. Catches within the Arua block declined but only to ~1 tsetse/trap/day until December 2012.

**Fig 7:**
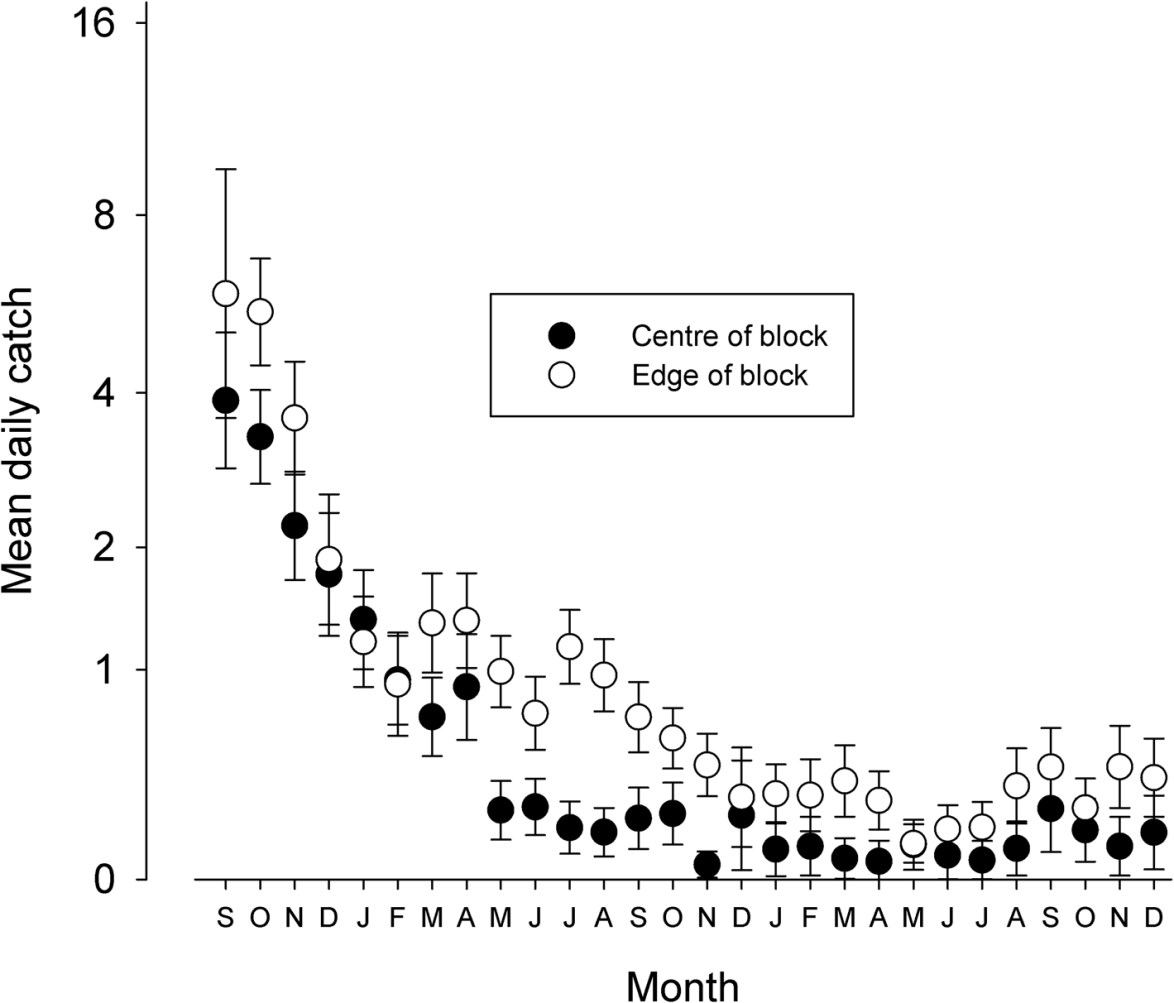
Catch of tsetse from traps at Centre or Edge of Phase 1 blocks. Mean daily catches from traps (±95%CI) at the Centre or Edge of an intervention block; data pooled for all intervention blocks.

As expected, targets had a more marked effect on the apparent abundance of tsetse at the centre of an intervention block (Fig 7) because of re-invasion along the river. This effect is demonstrated by comparing the mean catch of traps at the centre (‘Centre traps’) and the edge (‘Edge traps’) of the five blocks for each month. The results (Fig 7) show that at the beginning of the trial, the mean catch of tsetse from traps at the edge of the blocks was greater than that from the centre. When targets were deployed, catches from both groups declined at similar rates until May 2012 when the catches from Edge Traps stabilized at about 1 fly/trap/day whereas the catch from Centre Traps continued to decline to about 0.2 tsetse/trap/day. Hence by mid-2012 there was a significant difference in the catches of the two groups. From December 2012, targets were deployed across the trial area and the catch of Edge traps decreased to the same level as Centre traps. By June 2013 onwards, mean catches from Edge and Centre traps were both consistently <0.5 tsetse/trap/day across the Phase 1 blocks.

#### Modelling the effects of targets on tsetse populations

**Fig 8:**
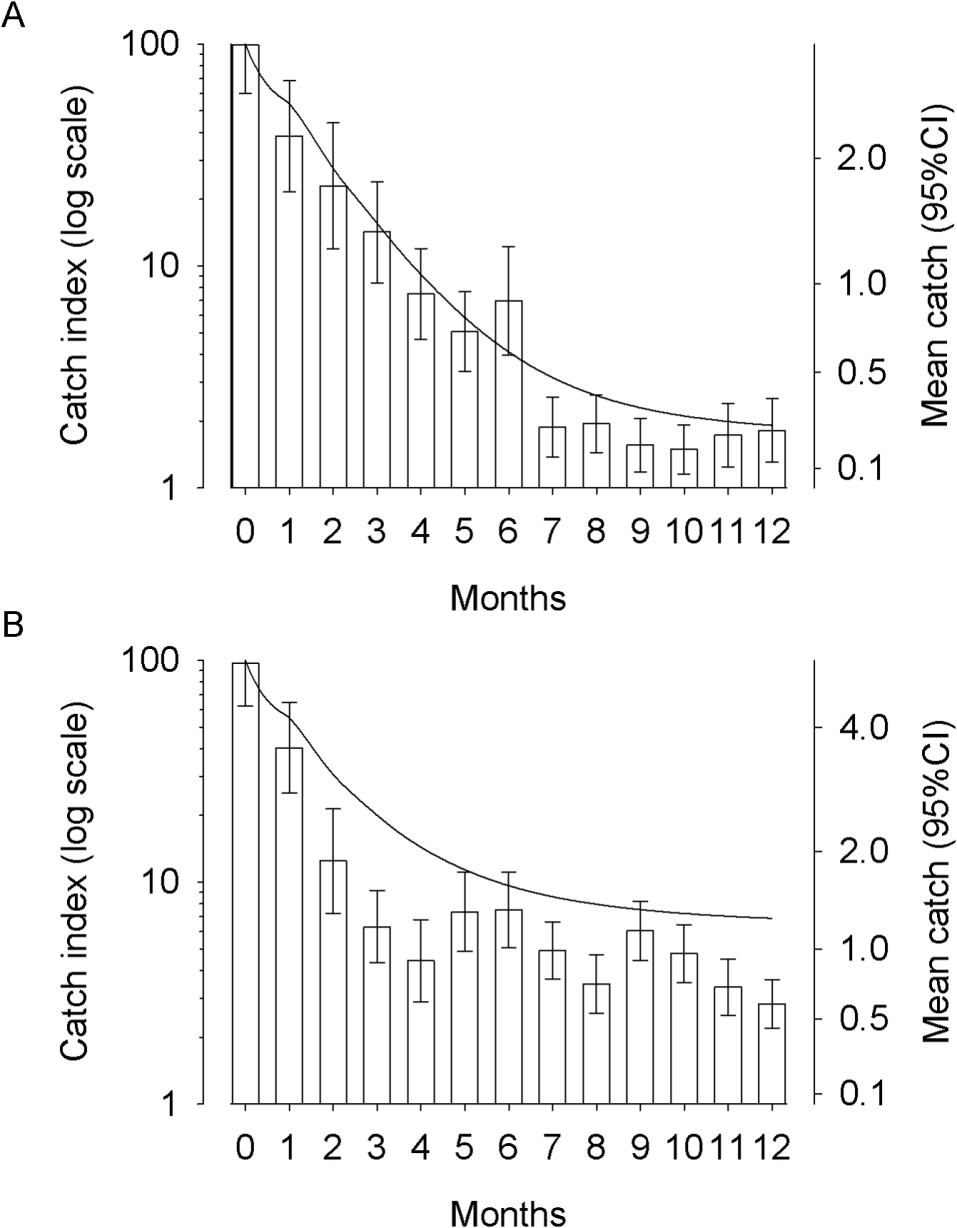
Predicted (lines, left y-axis) and observed (bars, right y-axis) decline in catch for (A) centre and (B) edge traps following deployment of targets in the small blocks.

The results from the island trials suggest that targets deployed at 50m intervals in riverine habitat imposed a mortality of 4%/day. Using this value, deploying targets along a 7km section of a river is predicted to reduce the density of tsetse by ~98% at the centre of the section and ~90% at its edge. The observed decline in catches from traps deployed at the centre of the block are in close accordance with the prediction, while the decline in catch from traps at the edge exceeded expectation (Fig 8). Unlike previous applications of Tsetse Muse [25,29], we did not vary the natural mortality of adults, pupae and eggs/larvae between cells but rather allowed that all sections of the river are equally suitable. This assumption provides a conservative estimate of the impact of targets. The greater-than-expected decline in catches from traps deployed at the edge of the intervention block is probably because blocks located nearer the sources of a river (i.e., Inve and Ayi) were subject to a lower level of re-invasion from upstream (ie river sections with poorer riverine vegetation), and hence the overall invasion pressure is reduced. Taking the results from the islands and the river together suggest that tiny targets deployed at these densities are imposing a daily mortality of ~4%–6%.

**Fig 9:**
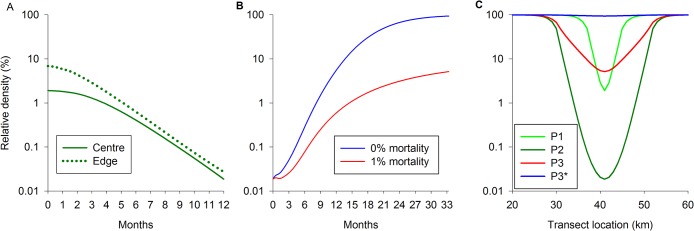
Predicted effect of targets on relative densities of tsetse. (A) Predicted density of tsetse from Centre and Edge positions following expansion of operational area from 7km (Phase 1) to 21km (Phase 2). (B) Rebound of tsetse at Centre following removal of targets or reduction in mortality imposed. (C) Relative density of tsetse along simulated river at the completion of Phases 1(P1) and 2 (P2) and rebound in tsetse when target density is reduced (P3) or targets are removed.

In Phase 2, the operational area expanded from the original 7 x 7 km blocks to cover an area of ~500 km^2^. Simulations of the expansion in the operational area predict that the density of tsetse at the edge and centre of the original (7x km) blocks will converge and decline to <1% of the original (pre-intervention) density after 12 months. The predicted convergence and decline is in accordance with the observed change in catch in May-June 2013 following the completed deployment of Phase 2 targets in January 2013 (Fig 9A and 9B).

#### Modelling impact of future operations

If targets are removed–or not maintained—at the end of Phase 2 then the population will recover to pre-intervention levels within ~1000 days (Fig 9B). However, if targets are maintained sufficiently to impose a daily mortality of, say, 1%/day, then the density of tsetse at the centre of the intervention area recovers to only 5% of its carrying capacity after 1,000d (Fig 9B). Changing the intensity of control not only alters the relative density of tsetse at the centre of the operation but also the surrounding area, as illustrated by Fig 9C. At the end of Phase 1 relative density was reduced by >90% across 5km of the operational area. Expansion of the operational area in Phase 2 increased the number of cells with >90% control to 19km. Reduced control (i.e., 1% mortality) allowed tsetse to reinvade and the area with >90% control is halved to 9km; >80% control is achieved in 13km. If control ceased completely then all the gains are lost with relative density in all cells being close to carrying capacity after 1,000d.

#### Costs of control

A full analysis of the costs of control are presented elsewhere [30]. We present here a preliminary account of the data to assist in the discussion which follows. The costs of control were broken down into five sub-activities; preliminary tsetse monitoring, sensitization of local populations, target deployment, monitoring of fly numbers and target maintenance. The headline figures for these costs are given in Table 1.

**Table 1:** Costs of control.

Preliminary Survey	Sensitization	Target cost and deployment	Monitoring	Target maintenance	Office maintenance	Total
5	16	29	9	17	9	85

An in depth economic analysis of costs of control operations in Uganda is presented in [30]. These are the estimated headline figures in US$ per km^2^ for a streamlined control operation covering 250 km^2^. The costs are in US$ per km^2^ per year if the project is run entirely within the country without external, specialist assistance or a research component.

Changes in attractiveness of field exposed tiny targets for flies is shown in Table B in S1 Text. The effective killing power of field exposed tiny targets is shown in Fig A in S1 Text.

## Discussion

Until very recently WHO advocated case detection and treatment as the only control method necessary for controlling Gambian HAT stating that for vector control “an ideal methodology easily accessible to the people at risk still does not exist” [31]. We agree that active case detection and treatment has proved an effective intervention against HAT outbreaks in many settings often bringing case loads down to levels where the local health system can cope. In addition, treatment is necessary if those already infected are not to die. But there is considerable doubt if case detection and treatment on its own can actually eliminate the disease. Most worryingly, it has been demonstrated that HAT detection rates during active case detection may be lower than 50% [6,32]. This means that many infected individuals will not be treated, even after repeated screenings, but will remain a source of infection in the community, sometimes for a long time before they die [33,34]. In addition, while the current accepted belief is that Gambian sleeping sickness is an anthroponosis *T*. *b*. *gambiense* has been found in several animals other than humans [35–37]. It is still unknown if non-humans are important reservoir hosts or not. If they are then vector control would become essential for the elimination of Gambian HAT as it already is for Rhodesian HAT [9]. Very recently WHO has acknowledged the potential benefit of incorporating vector control in some settings [38]. This reflected the findings of modellers who much earlier had predicted that vector control was necessary in foci with high transmission rates and that it would be highly effective in other situations as well [10]. Here we discuss an affordable and effective vector control system suitable for use at the level of the HAT focus. We advocate its use on a broad scale and in close partnership with case detection and treatment systems.

Odour-baited targets have been widely used to control savannah tsetse in east and southern Africa but not for riverine tsetse in Central and West Africa. The general perception is that the relatively poor response of riverine tsetse to host odours [39,40] [41] means that baits need to be deployed at densities 10x greater than that required for savannah tsetse (i.e., 40 vs. 4 targets/km^2^). We demonstrate here that this general perception is not correct. Riverine tsetse usually have restricted distribution in the habitat, often confined to river banks. This means that the deployment of baits can also be restricted to these areas. Hence in Uganda, targets were deployed at high densities along the rivers (~20 targets per linear km) but nowhere else. This meant the overall density of tiny targets was ~5.7 targets/km^2^ and not the 40/km^2^ often mentioned in the literature for other tsetse killing devices. To state this in another way the entire 500km^2^ covering two HAT foci in N. Uganda was successfully treated (including replacements) with ~ 4,208 tiny targets per year. Such deployments reduce the density of tsetse >90% within three months. The reduction is more marked and sustained for relatively isolated populations such as the islands on Lake Victoria but even on the edge of controlled areas where reinvasion inevitably occurs, the relative abundance of tsetse was reduced by >85%.

What are the costs of such a vector control operation? The cheapest means of controlling tsetse is to treat cattle with insecticides effective against tsetse [42]. However, this requires cattle to be present at densities sufficient to provide an important part of the diet of tsetse. The minimum density of cattle required will depend on the availability of alternative wild hosts but is likely to be in the region of 10 cattle/km^2^. This precludes the use of this technique in many Gambian HAT foci [11,43]. Artificial baits like those used here are the next cheapest option. We have undertaken an economic analysis of the costs of the control operation in N. Uganda [30]. Traditionally such control required large teams and lorries to transport and maintain bulky and expensive biconical traps and their supports. In sharp contrast tiny targets are cheap: thirty can be easily placed in a single backpack and can be deployed along the riverbank by one man with minimal logistical support. Consequently, comparable control costs have dropped by >80% from US$556 per km^2^ per year [42] to US$85.4 (Table 1). So the cost of treating these two HAT foci covering 500km^2^ by an in country tsetse control programme is US$42,700 per year. To place this in context, using W.H.O. figures from 1998, an active screen covering 100,000 people cost in the region of US$150,000 at that time (this excludes treatment costs estimated at US$1M for 5,000 people). The Ugandan national census suggests the population density of the Arua/Maracha/Terego area is 500 people per km^2^. If we use a compound inflation figure of 45% from 1998 to 2014 the estimated cost for a single round of active case detection (excluding treatment), covering 80% of the population in the HAT foci, is therefore US$433,333. One year of vector control organised within the country, which can completely stop HAT transmission, would cost US$42,700. The case for adding this refined method of vector control to case detection and treatment is strong. We outline how such a component could be organised.

**Fig 10:**
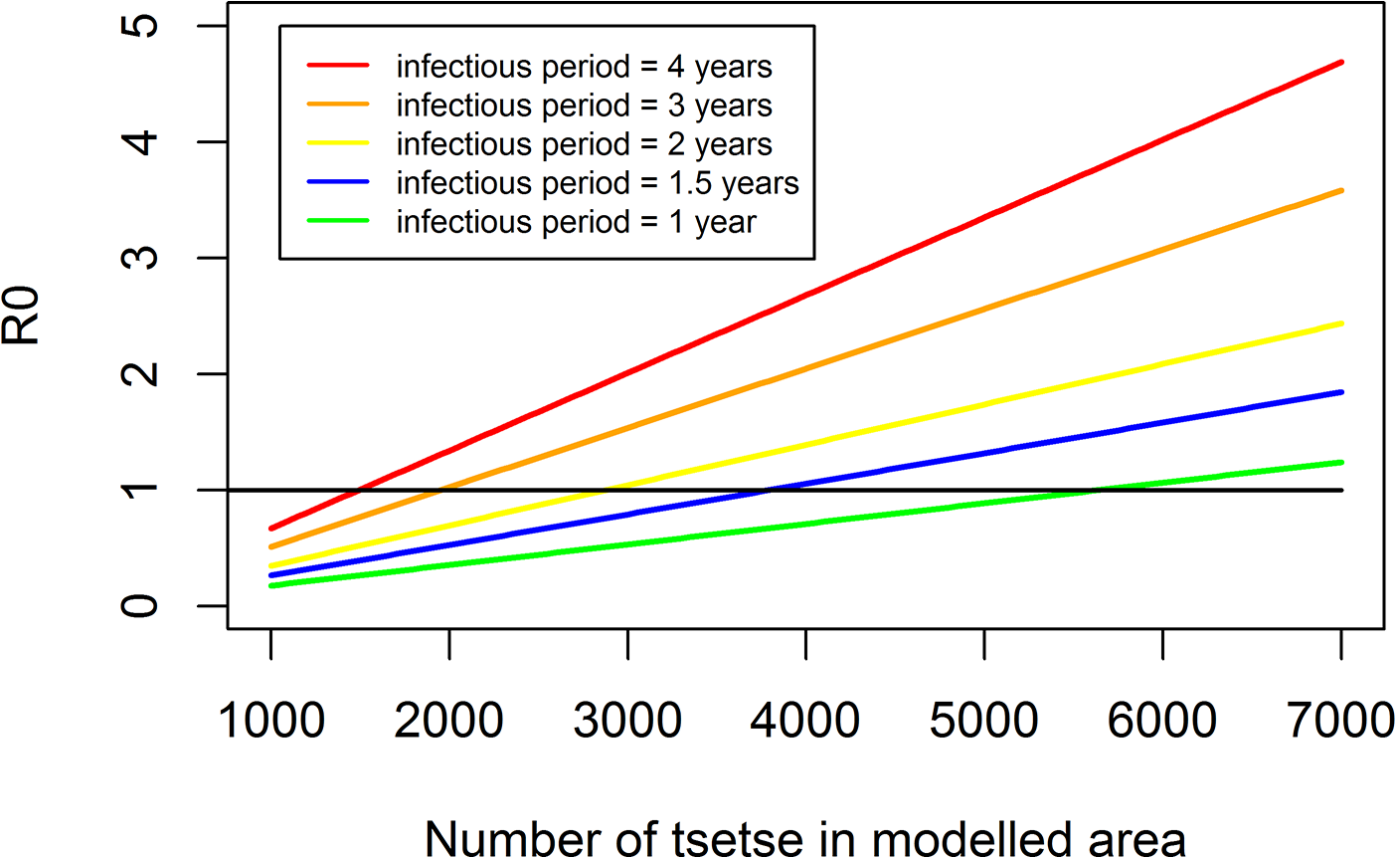
Relationship between Ro (y-axis) and numbers of tsetse (x-axis) for settings where the average infectious period is 1–4 years. To obtain an estimate of the level of tsetse control required to stop transmission we have re-arranged a published model [10]. The average infectious period is usually accepted as 3 years and so we can see that a reduction in tsetse numbers of approximately 72% is required to drive R0<1.

Would a tsetse control operation such as we have described here have an impact on HAT transmission? To understand more clearly what level of tsetse control would be required to stop HAT transmission completely we have re-arranged a published HAT transmission model [10]. The normal infectious period for Gambian sleeping sickness is three years [44]. From Fig 10 we can see that under those conditions 72% reductions in tsetse numbers will stop HAT transmission. We have been routinely achieving ~90% reductions (Figs 3, 4 & 6). The epidemiological data presented from Guinea in an accompanying paper in this edition demonstrates that an average 80% vector control had a significant impact on HAT transmission [45]. If tsetse control operates in tandem with active case detection and treatment programmes we can expect average infectious periods to fall. Under those conditions even more modest levels of tsetse control interrupts transmission (Fig 10). It is important to realise that the type of programme described here does not aim to eradicate tsetse, merely to hold the tsetse population levels below the threshold required for HAT transmission for a prescribed period of time. Once tsetse control ceases fly populations will gradually return. If *T*. *b*. *gambiense* is no longer in the focus this does not present a problem. It is clear that halting HAT transmission does not require the elimination of tsetse and, in support of this, historical foci of HAT with remaining tsetse populations are well known [46] reminding us of the classic ‘anophelism without malaria’ debates of a century ago.

Clearly, under ideal conditions, tsetse control operations should remain in place until all risk of transmission ceases. Using published data [44] survival in stage 1 appears to decay exponentially (daily rate = 0.0019; mean stage 1 duration = 526d (95%CI 357 to 833)), and stage 2 is 500d (95%CI 345 to 769). Consequently the average duration of infection is at least 3 years and the upper 95%CI is 833+769 = 4 years. Modelling is clearly required to provide a better estimate but we estimate that vector control will be required for about five years.

Our recommendation is for target deployment to commence at the very start of the dry season. Typically in Uganda this lasts about three months by which time the control activities will have reduced the tsetse population by >80% (Fig 8). Once the rains recommence some loss of targets from flooding will occur. The level and location of loss is determined in the maintenance phase and targets are replaced as necessary. The effective lifetime of the targets used in this study was not limited by decreasing attractiveness of the tiny targets caused by weathering (see S1 Text). However decreasing performance of the insecticide limited their effectiveness to 6–8 months (Fig A in S1 Text). A longer effective life might be obtained by changing the dose of insecticide applied to the netting or the physico-chemical attributes of the target material [47] [48].

How would a control operation such as we describe above be organised in practice? The essential components of the programme are 1. The production of GIS-generated maps of the area with particular emphasis on rivers and access; 2. A preliminary entomological survey to determine fly densities enabling planning of the deployment phase; 3. Sensitization of the local community [49]; 4 Training followed by target deployment; 5. Target maintenance and fly monitoring activities for fine scale management of vector control activities. We would suggest that control be organised at the level of the HAT focus ideally using personnel from health or vector control structures already working in the focus. For example, we worked closely and effectively with womens' groups and local government at the District-Parish levels [49]. The technical skill required for target deployment itself is minimal but the programme does require effective management. Our experience suggests that managerial support and training would need to be provided by an external team at the beginning of an intervention. Modelling suggests that once an epidemiologically-significant (>75%) level of control is achieved in year one then even if control efforts are less effective (as little as a quarter) in subsequent years the fly population will not recover completely (Fig 9). This suggests that external support to the local team in year one could be rapidly tapered off thereafter with some confidence that vector control will continue to be effective.

In summary we believe that given the incomplete coverage achieved by case detection and treatment programmes the development of cost-effective tiny target technology makes a strong case for the inclusion of vector control alongside case detection and treatment in operations against Gambian HAT.

## Supporting Information

S1 Text Supporting materials. (DOCX)
