# Supplementary material for: Tsetse Control and Gambian Sleeping Sickness; Implications for Control Strategy
Source: PLoS Negl Trop Dis. 2015 Aug 12;9(8):e0003822. doi: 10.1371/journal.pntd.0003822 (PMC4580652; doi:10.1371/journal.pntd.0003822)
Supplement: S1 Text — (DOCX) [file pntd.0003822.s001.docx]

**Supplementary material**

**S1. Simulating the control of tsetse populations using Tsetse Muse**

The Tsetse Muse model used in the present paper is described by [1] and can be downloaded for inspection and use at [www.tsetse.org](http://www.tsetse.org). Those aspects of the model related to present simulations of non-isolated populations are summarised below and in Table A. Simulations of isolated populations were the same except that it was not necessary to envisage separate segments of habitat between which flies could move.

**Population**

The adult tsetse population before intervention was stable at carrying capacity, comprising 2500 adult males and 5000 adult females per km^2^ of habitat [2], and was considered to occupy a strip of uniformly good habitat 60km long, in which the numbers in each 1km long segment were recorded each day. The total population of each segment was separated into pupae and adults of each sex. Adult females comprised virgins and mated individuals that started their larval cycle at the normal time, and also females mated too late to join the first larval cycle and so were waiting to join the next available cycle. Each component of the population in each segment of habitat was tracked deterministically in its own life table produced in a Microsoft Excel spreadsheet. For convenience, all flies were considered to occur at the centre of the segment they occupied, and the lifespan of adults was limited to 89 days for males and 178 for females, according with the evidence [3] that females live about twice as long as males. In each cell of each table the Excel formulae performed the following procedures to cater for movement, death and development as each fly became a day older.

**Movement**

A certain proportion of the adult flies in the life table of each segment of habitat transferred to the life tables of the adjacent segments, to give the required rate of average daily displacement. The rate of movement was age-dependent, to accord with the fact that flight musculature takes a week or so to develop [4], and that wings become badly damaged in very old flies [5]. For males the average displacement was 100m on the first day of adult life, increasing linearly to 300m/day at age 10 days. This rate was maintained until age 25 days and then declined linearly to 200m/day at maximum age. For females the rates were the same except that the 300m/day maximum lasted longer, from age 10-50 days, and the subsequent decline to 200m/day at maximum age was slower since the maximum age was greater. These rates of movement are consistent with field data [6]

**Deaths**

Tsetse occurring in each cell after daily movement were subject to deaths. Natural death rates of adults were age dependent, and greater for females than for males [2]. In contrast to previous applications of the model [1,7,8], in the present application we simplified the inputs by assuming that natural death rates did not vary between adjacent segments of habitat, but rather allowed that all segments were equally and highly hospitable. This assumption ensured a conservative estimate of the impact of targets. The natural rates for each sex were high on the first day of life. Thereafter the rates declined linearly to be minimal at age 10 days, at which time the rate as a proportion of the initial rate was a fifth for males and a tenth for females. Subsequently, the rates increased linearly to be treble the initial rate at maximum age and were 1.00 when the maximum lifespan was exceeded. Although natural deaths of pupae and eggs/larvae were considered to occur each day it was simplest to apply all daily deaths due per period at the start of the pupal period or the end of the larval period, since the age structure of the pupal and uterine populations was irrelevant.

All natural death rates of adults, pupae and eggs/larvae were density dependent, consistent with field evidence for the density dependence of rates of parasitism and predation [9] and feeding success [10]. It was assumed that with a reduction in density the death rates declined linearly to a minimum of three-quarters of the stable rate at 10% of stable density. For adults and eggs/larvae of either sex the reference density was the total density of adult males and females. For pupae it was the total live pupae of either sex.

Adult death rates due to targets were assumed to be the same for each sex and age class and were imposed, in those segments of habitat that contained targets, on adults that survived natural death. The percentage was varied but often set at 4%, the theoretical minimum that will lead to the elimination of a tsetse population [3,11].

**Development and reproduction**

Rates of development of pupae and the timing of the first and subsequent larval periods were for temperatures of 25^o^C [3,12]. Transfer of flies from pupae to new adults, the passage of adults to sexually maturity, and any mating, occurred at the start of each day before movement and deaths. Transfer of larvae to pupae was performed at the end of the day, after deaths. The daily probability of a mature virgin female mating with a mature male, when the density of such males is 1/ km^2^, was taken as 0.1, associated with daily probability of not mating of 1-0.1 = 0.9. Hence, the actual daily probability (*q*) that a mature virgin female in any segment of habitat would not mate was 0.9*^a^*, where *a* is the actual density of mature males per km^2^ within the segment of habitat. The actual probability that the mature virgin female would mate was 1-*q*.

**Management of calculations**

The procedures of the model were managed by Visual Basic for Applications. The initial tasks were to establish the actual death rates of adults of each sex on the first day of life, from which all other death rates of all age classes of each sex could be deduced from the adopted pattern of age-dependent deaths, above. These tasks were performed by Excel's Solver facility which was programmed first to find the death rate of adult females needed to produce a stable female population when males were assumed sufficiently dense to ensure that all virgin females were mated on the day of their maturation. Next, Solver found the death rate of adult males that was required to produce the adopted sex ratio of the adult population. From these parameters and the other inputs it was possible to establish the number of tsetse in each cell of the life table of each component of the stable tsetse population in each segment of the habitat prior to intervention.

To start the simulated intervention, the stable pre-intervention numbers of tsetse were put into all life tables of all segments, and Excel's calculation mode was set to iterate. At each iteration, regarded as the passage of a single day, a wave of calculations started at the top of the worksheet and passed down. The wave travelled first through preliminary calculations of the density-dependent death rates applicable to the tsetse densities in each segment of habitat on the day being simulated. This density was that displayed in the life tables lower on the sheet, where the wave of calculations had not yet reached, so that the density displayed there then was that for the end of the previous day and hence for the start of the day being simulated. When the wave of calculation went lower on the sheet, to move through the life tables, it advanced the age of flies by one day, i.e., shifted them up to the next daily age class of the life tables, and performing in that process all due movements, deaths, development and transfers between population components. At the bottom of the sheet the number of new-born larvae were added to the pupal section of the life tables, as pupae of age 0 days. After simulation of the required number of days of intervention, the population remaining in all cells of all life tables was copied and pasted to the record of outputs, prior to resetting Excel to its normal calculation mode.

**References**

1. Vale GA, Torr SJ. User-friendly models of the costs and efficacy of tsetse control: application to sterilizing and insecticidal techniques. Med Vet Entomol. 2005; 19: 293-305.

2. Leak S. Tsetse Biology and Ecology, Wallingford, CABI Publishing; 1998.

3. Hargrove JW. Tsetse population dynamics. In: Maudlin I, Holmes PH, Miles MA, editors. The Trypanosomiases. Wallingford: CABI publishing; 2004. pp. 113-137

4. Bursell E, Kuwengwa T. The effect of flight on the development of flight musculature in the tsetse fly (*Glossina morsitans*). Entomol Exp et Appl. 1972; 15: 229-237.

5. Jackson CHN. An artificially isolated population of tsetse flies (Diptera). B Entomol Res. 1946; 37: 291-299.

6. Rogers D. Study of a natural population of *Glossina fuscipes fuscipes* Newstead and a model of fly movement. J Anim Ecol. 1977; 46: 309-330.

7. Kgori PM, Modo S, Torr SJ. The use of aerial spraying to eliminate tsetse from the Okavango Delta of Botswana. Acta Trop. 2006; 99: 184-199.

8. Torr SJ, Vale GA Is the Even Distribution of insecticide-treated cattle essential for tsetse control? Modelling the impact of baits in heterogeneous environments. PLoS Negl Trop Dis. 2011; 5(10): e1360.

9. Rogers DJ. Estimation of rates of predation on tsetse. Med Vet Entomol. 1990; 4: 195-204.

10. Vale GA. Feeding responses of tsetse flies (Diptera: Glossinidae) to stationary hosts. B Entomol Res. 1977; 67:635-649

11. Hargrove JW. Tsetse: the limits to population growth. Med Vet Entomol. 1988; 2: 203-217.

12. Glasgow JP. The distribution and abundance of tsetse. Oxford: Pergamon ;1963

Table A. Features of the simulated population at stable carrying capacity

| **Feature** | | | **Males** | **Females** | **Source** |
| --- | --- | --- | --- | --- | --- |
| Adults per km^2^ of habitat | | | 2500 | 5000 | [2] |
| Mean adult age | | | 24 | 44 | M [3] |
| Pupal period | | | 28 | 26 | [3] |
| Age at sexual maturity | | | 5 | 3 | [12] |
| Age at production of first larva | | | - | 16 | [3] |
| Interlarval period | | | - | 9 | [3] |
| Maximum adult lifespan | | | 89 | 178 | [12] |
| Death rates | Pupae per pupal period | | 25 | 25 | [3] |
|  | Eggs/larvae per larval period | | 5 | 5 | [3] |
|  | Adults per day | Average | 6.14 | 3.07 | M [3] |
|  |  | First day | 14.21 | 13.62 | M [3] |
|  |  | Young adult | 2.84 | 1.36 | M [3] |
|  |  | Last day | 8.52 | 4.09 | M [3] |
| Average daily displacement, metres | | | 300 | 300 | [6] |

All times and ages are in days. Death rates are the natural rates quoted as percents; they varied with age, as discussed in the text. Young adults were males 10-15 days old and females 10-50 days old. Average daily displacement shown refers to young adults. It varied according to age, as indicated in the text. Where M occurs in the source list, the exact value was calculated by the model itself but is within the range expected from published data.

**S2: Effective life of tiny targets: effects of weathering on attractiveness**

There was no significant difference in the catch of tsetse from e-targets made using cloth and netting taken new and old ‘tiny targets’ produced by Vestergaard-Frandsen or a Standard target (Table B). The cloth and netting panels from tiny targets were made from blue polyester and black polyethylene, as against phthalogen blue cotton and fine black polyester netting for the Standard target. The Standard target has been used in previous studies of various coloured targets (Lindh *et al*., 2012).

Table B. Detransformed mean daily catch (detransformed mean ±95% CI in brackets) of *G. f. fuscipes* from Standard, New and Old e-targets.

| Target | Age comparison | | |
| --- | --- | --- | --- |
|  | 6 months (18 replicates) |  | 12 months (36 replicates) |
| Standard^1.^ | 26.6 |  | 13.4 |
|  | (1.44±0.065) |  | (1.16±0.071) |
| Not weathered^2.^ | 22.5 |  | 10.6 |
|  | (1.37±0.091) |  | (1.06±0.062) |
| Weathered^3.^ | 23.4 |  | 11.8 |
|  | (1.39±0.087) |  | (1.11±0.068) |
| *P* | *n.s.* |  | *n.s.* |

1. Standard target: pthalogen blue cotton and polyester netting, not weathered.

2. Not weathereed target: blue polyester and black polyethylene netting, not weathered.

3. Weathered target: blue polyester and black polyethylene netting, weathered for 6-12 months.

**Reference**

Lindh JM, Goswami P, Blackburn RS, Arnold SEJ, Vale GA, et al. (2012) Optimizing the Colour and Fabric of Targets for the Control of the Tsetse Fly Glossina fuscipes fuscipes. Plos Neglected Tropical Diseases 6.

**S3: Effective life of tiny targets: effects of weathering on insecticide performance**

Cloth and netting from tiny targets was bioassayed by exposing wild caught (i) female *G. pallidipes* from Zimbabwe or (ii) *G. f. fuscipes* from Mbita to netting and cloth from tiny targets weathered for 0-8 months. Bioassays were carried out at, respectively, Rekomitjie Research Station in Zimbabwe or ICIPE’s Thomas Odhiambo Campus at Mbita, Kenya. See Torr (1985) for further details of methods.

Knockdown of tsetse exposed to untreated netting or cloth was <4% for all assays. The results for netting show (Fig. A) that exposing tsetse to new (<1 month exposure) produced a 100% knock down. Thereafter the knockdown was variable and declined to <50% after six months. For tsetse exposed to cloth, knockdown exceeded 75% consistently for eight months when testing ceased (Fig. A).

Fig. A Percentage knockdown of tsetse 2 h after exposure to netting (A) or cloth (B) from tiny targets.

Each dot represents the percentage knockdown from a bioassay of a single sample using ~30 tsetse (median=30, range 19-34). Bars indicate mean percent knockdown from all assays conducted for each month. Where several samples produced the same knockdown then only one dot is visible, but the total number of samples tested is shown in text within each bar.

**Reference**

Torr SJ (1985) The susceptibility of *Glossina pallidipes* Austen (Diptera: Glossinidiae) to insecticide deposits on targets. Bulletin of Entomological Research 75: 451-458.
